# Abdominal wall hernia in cirrhotic patients: emergency surgery results in higher morbidity and mortality

**DOI:** 10.1186/s12893-015-0052-y

**Published:** 2015-05-21

**Authors:** Wellington Andraus, Rafael Soares Pinheiro, Quirino Lai, Luciana B.P Haddad, Lucas S Nacif, Luiz Augusto C D’Albuquerque, Jan Lerut

**Affiliations:** Digestive Organs Transplant Unit, Department of Gastroenterology, University of Sao Paulo School of Medicine, Sao Paulo, Brazil; Department of Hepatic Surgery and Liver Transplantation, Azienda Universitario-ospedaliera Pisana, Pisa, Italy; Starzl Unit of Abdominal Transplantation, University Hospital of Saint Luc, Université Catholique of Louvain, Brussels, Belgium

**Keywords:** Abdominal hernia, liver cirrhosis, ascites

## Abstract

**Background:**

Patients with cirrhosis have a high incidence of abdominal wall hernias and carry an elevated perioperative morbidity and mortality. The optimal surgical management strategy as well as timing of abdominal hernia repair remains controversial.

**Methods:**

A cohort study of 67 cirrhotic patients who underwent hernia repair during the period of January 1998-December 2009 at the University Hospital of Sao Paulo were included. After meeting study criteria, a total of 56 patients who underwent 61 surgeries were included in the final analysis. Patient characteristics, morbidity (Clavien score), mortality, Child-Turcotte-Pugh score, MELD score, use of prosthetic material, and elective or emergency surgery have been analysed with regards to morbidity and 30-day mortality.

**Results:**

The median MELD score of the patient population was 14 (range: 6 to 24). Emergency surgery was performed in 34 patients because of ruptured hernia (n = 13), incarceration (n = 10), strangulation (n = 4), and skin necrosis or ulceration (n = 7). Elective surgery was performed in 27 cases. After a multivariable analysis, emergency surgery (OR 7.31; p 0.017) and Child-Pugh C (OR 4.54; p 0.037) were risk factors for major complications. Moreover, emergency surgery was a unique independent risk factor for 30-day mortality (OR 10.83; p 0.028).

**Conclusions:**

Higher morbidity and mortality are associated with emergency surgery in advanced cirrhotic patients. Therefore, using cirrhosis as a contraindication for hernia repair in all patients may be reconsidered in the future, especially after controlling ascites and in those patients with hernias that are becoming symptomatic or show signs of possible skin necrosis and rupture. Future prospective randomized studies are needed to confirm this surgical strategy.

## Background

**Fig. 1:**
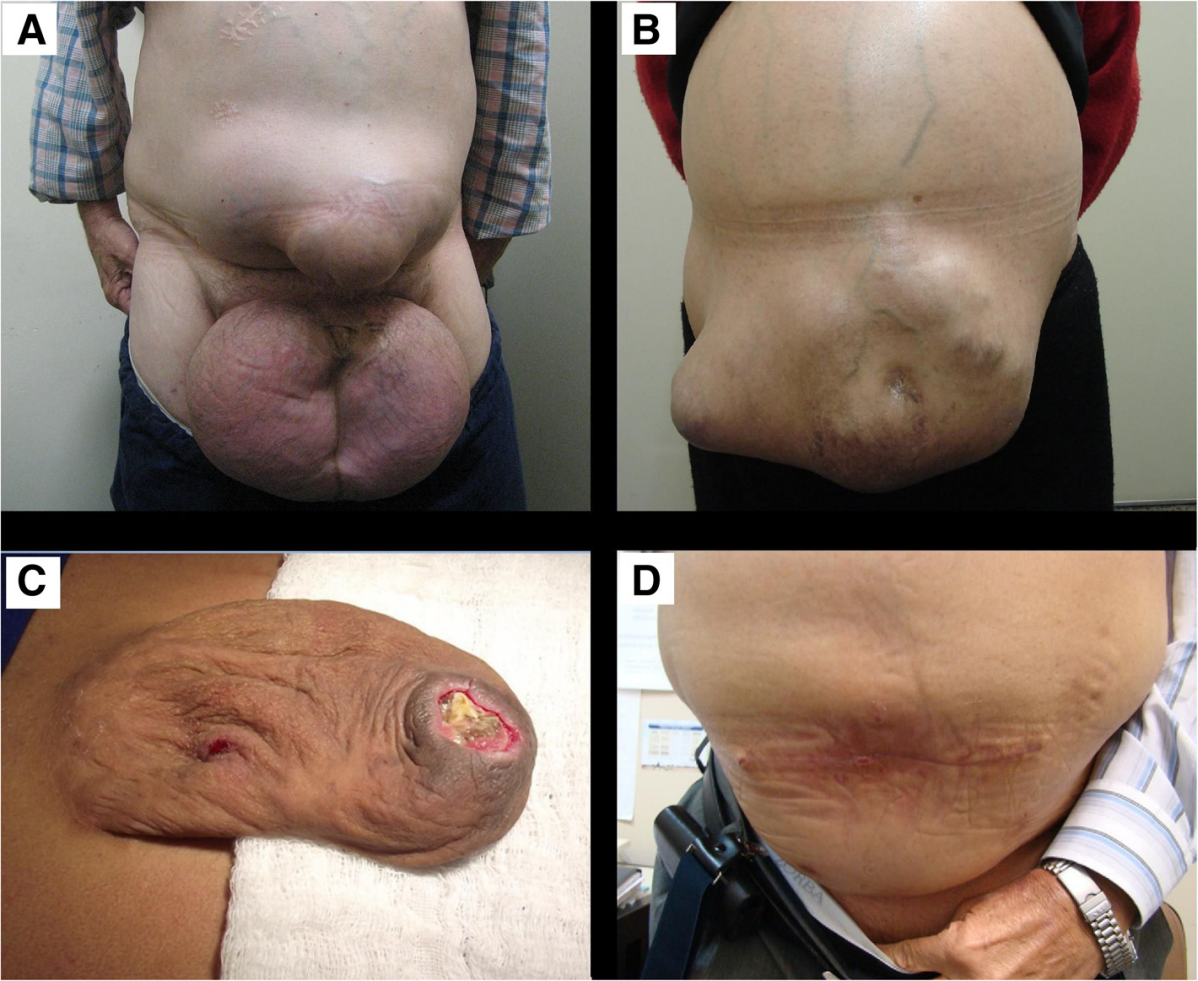
Representation of cirrhotic patients with abdominal wall hernia. **a**-Male patient with bilateral inguinal and umbilical hernia. **b**-Female patient with enormous umbilical hernia. **c**-Ischemic skin ulceration rupture of huge umbilical hernia in a cirrhotic patient. **d**-Child B patient with uncontrolled ascites after one-year follow up after umbilical hernia repair

The incidence of abdominal wall hernias in cirrhotic patients is as high as 20 % and in cases of major ascites this number may increase up to 40 % [1, 2]. Several factors such as increased abdominal tension due to the presence of tense ascites, malnutrition, and worsening muscle wasting are major risk factors for the development of abdominal hernias in these patients [3]. Moreover, due to the presence of increased surgical risk factors in cirrhotic patients, high perioperative morbidity and mortality are often encountered [4]. It is commonly accepted that surgical correction of abdominal wall hernias in cirrhotic patients should not be done electively. The more conservative ‘wait and see’ policy is frequently advocated because of high perioperative morbidity and mortality rates [5–7] . Consequently, patients with refractory ascites presenting with an abdominal hernia are generally treated conservatively, a strategy frequently leading to great discomfort and risk of life threatening complications such as incarceration and skin rupture (Fig 1). Ideally, abdominal wall hernias should be repaired in chronic liver disease patients during or after successful liver transplantation (LT). However, the interval between the development of a significant hernia and LT is often very long due to ever increasing transplant waiting times [5]. Some studies have shown risks of elective surgery in cirrhotic patients are not prohibitive even in the presence of refractory ascites or advanced cirrhosis under the condition that such procedures are performed in a highly experienced liver centre [8–10].

The aim of our study is to analyse the characteristics of unselected cirrhotic patients who underwent hernia repair in a tertiary referral centre. The secondary end-point is to investigate risk factors related to post-operative morbidity and 30-day mortality.

## Methods

This retrospective study was conducted on all patients with documented cirrhosis who underwent hernia repair at the Department of Digestive and Liver Transplant Surgery at the University of Sao Paulo Medical School (FMUSP) from January 1998 to December 2009. A total of 67 patients underwent 74 surgeries; however, 56 patients who underwent 61 surgeries were included in the final analysis. Eleven patients were excluded due to lack of medical records (one patient), incomplete laboratorial evaluation (eight patients), and loss of follow-up (two patients). Cirrhosis was confirmed in all patients by liver laboratory tests and confirmed with liver biopsy, imaging, or intraoperative findings. Abdominal wall hernias were diagnosed by physical examination and ultrasound and/or CT scans were occasionally used during the diagnostic work-up when necessary.

Patient characteristics are displayed in Table 1. Clinical data included poorly controlled ascites before surgery, length of intensive care and hospital stays, as well as morbidity and 30-day mortality rates. Morbidity was classified according to the Clavien-Dindo classification and class III to V events were considered to be major complications. Post-operative mortality was considered up to 30-days after surgery; after this period, mortality was strictly related to cirrhosis complications rather than complications of the surgical procedure. A longer follow-up period was employed to evaluate hernia recurrence, which were diagnosed by physical examination and in difficult cases ultrasound and/or CT scan were used to confirm the diagnosis.

**Table 1:** Demographics, hernia, and underlying liver disease characteristics and post-operative course in the entire cohort and in the two subgroups of patients underwent elective or emergency operations

Variables	Entire cohort (n = 61)	Elective cases(n = 27)	Emergency cases(n = 34)
Age (range in years)	52 (44–62)	51 (45–60)	53 (43–65)
Male gender (%)	46 (82.1)	20 (87.0)	26 (78.8)
Mean BMI^a^ (range)	25 (16–36)	28 (19–34)	24 (16–36)
Poorly controlled ascites (%)	25 (41)	2 (7.4)	23 (67.6)
CTP^b^ (%):A	19 (31.1)	16 (59.2)	3 (8.8)
B	29 (47.5)	9 (33.3)	20 (58.8)
C	13 (21.4)	2 (7.5)	11 (32.4)
Mean Pre-operative MELD^c^ (range)	14 (8–22, 24, 25)	12 (8–21, 24, 25)	16 (8–22, 24, 25)
Hernia surgery (%):umbilical	39 (54.9)	9 (25.7)	30 (83.3)
inguinal	23 (32.4)	19 (54.3)7	4 (11.1)
other	9 (12.7)	(20)	2 (5.6)
Combined surgery (%)	10 (17.9)	8 (34.8)	2 (6.0)
Ascites leak (%)	13 (23.2)	0 (−)	13 (39.4)
Incarceration (%)	10 (16.4)	0 (−)	10 (29.4)
Use of prosthesis (%)	24 (39.4)	16 (59.3)	8 (23.5)
Mean Hospital stay (range in days)	11 (3–33)	5 (3–14)	13 (6–33)
ICU^d^ stay (%)	22 (36)	4 (14.8)	18 (53)
Mean ICU^d^ stay (range in days)	7 (0–33)	2.5 (0–4)	9 (0–33)
Morbid Event (post-operative complication) (%)	26 (42.6)	4 (14.8)	22 (64.7)
Death (%)	11 (18)	1 (3.7)	10 (29.4)
Clavien-Dindo classification (%):	3 (4.9)x	1 (3.7)	2 (5.8)
I	6 (9.8)	1 (3.7)	5 (14.6)
II	2 (3.3)	0 (−)	2 (5.8)
III	4 (6.5)	1 (3.7)	3 (8.9)
IV	11	1 (3.7)	10 (29.4)
V	(18)		
Mean Post-operative MELD (range)	16 (13–21, 24, 25)	14 (11–15)	21 (15–30)

Abbreviations: ^a^BMI, body mass index; ^b^CTP, Child Turcotte Pugh; ^c^MELD, model for end-stage liver disease; ^d^ICU, intensive care unit

Combined surgery: two different sites of hernia repair in the same surgical procedure

Liver disease severity was documented by the Child-Turcotte-Pugh (CTP) classification and MELD score [11, 12]. All values were calculated at the moment of hernia repair.

Pre- and postoperative management of patients presenting with major ascites consisted of paracentesis with concomitant intravenous albumin substitution, nutritional support, and correction of coagulation disorders when indicated. Hernia repairs were performed with primary musculo-fascial closure and completed with the use of onlay prosthetic material (Prolene mesh) in certain cases. An emergency procedure was defined as a surgical hernia repair that occurred up to 12 h after the diagnosis of ascites leakage due ruptured hernia, incarceration with refractory pain, strangulation, and extensive skin necrosis or ulceration.

### Ethics

This study was approved by the Institutional Review Board of FMUSP.

### Statistical analyses

Categorical variables are reported as number of cases and percentages. Continuous variables are provided as medians and inter-quartile ranges (IQR). Two univariate logistic regression analyses were performed with the intent to investigate variables connected with post-operative morbidity and 30-days mortality.

All variables identified after univariate analyses with a p-value < 0.20 were considered for the construction of two conditional logistic regression models. Due to the small cohort of patients in this study, we determined the number of covariates necessary to adopt the multivariable model and assumed the following: N > 50 + m (N = number of cases, m = number of covariates) [13] . Goodness of fit in the models was tested with the Hosmer-Lemeshow test. Risk predictions are reported as a p-value, odds ratio (OR), and 95 % confidence intervals (95 % CI). A p-value < 0.05 indicates statistical significance. The backward conditional method was used for constructing models with the intent to include only main effects. Statistical analyses were performed with SPSS 19.0 (SPSS, Chicago, IL).

## Results

A total of 56 cirrhotic patients underwent 61 surgeries to repair 71 hernias. In 10 surgeries two different hernia sites were corrected (mainly inguinal and umbilical). Sequential surgical procedures were performed at least 6 months after the first procedure due large hernias in more than one site in 3 patients and due to hernia recurrence at the original site in 2 patients. In terms of hernia repair urgency, 34 (55.7 %) repairs were performed emergently and 27 (44.3 %) were considered elective procedures. The median pre-operative MELD score of the cohort was 14 (IQR 11–18) and numbers of patients with a score > 20 were similar between the patients who underwent emergency or elective operations (p 0.216). All patients had a minimal follow-up of two years after surgery. The median follow-up of the entire cohort was 27 months (range 1 to 115 months). The wide variation is due to mortality, considering that the median follow-up of the survivors was 52 months (range 24 to 115 months).

Patients underwent elective operations based upon individual assessment of preoperative risk and of the symptoms caused by the hernia. In terms of hernia type, 15 patients had 19 inguinal hernias, 9 had an umbilical hernia, 5 had an incisional hernia, 1 had an epigastric hernia, and 1 had a perineal hernia. Combined hernia repair (two different hernia repair sites during the same operation) was performed 8 times. Polypropylene mesh was used in 16 (59.3 %) patients. Two patients presented with refractory ascites after surgery.

Surgery was performed emergently because of hernia rupture (n = 13), incarceration (n = 10), strangulation (n = 4) and skin ischemia or ulceration (n = 7). Among patients requiring an emergency operation, 30 had an umbilical hernia, 4 had an inguinal hernia, and 2 had an epigastric hernia. Combined hernia repair was performed in 2 cases. Polypropylene mesh was used in 8 (23.5 %) patients and 23 of these patients presented with major ascites.

A total of 11 (19.6 %) patients died within 30 days after surgery. Ten patients (17.8 %) died after an emergency surgical procedure. All these patients had renal failure and three patients had a local complication including leaking ascites and wound infection. There was one patient who required a reoperation. In contrast to the emergency surgery group, only 1 (1.8 %) patient who underwent an elective hernia repair died because of intracranial haemorrhage, a condition not directly related to the surgery.

Long-term outcomes were not influenced by hernia repair in any case and 23 patients died due cirrhosis complications and 1 patient underwent LT.

Emergency patients also presented with a markedly higher number of peri-operative class III–V complications according to the Clavien-Dindo classification (44.1 % vs. 7.4 %).

Median hospital stay of the entire cohort was 11 days (IQR 4–19). Longer median hospital (13 vs. 5 days) and intensive care unit stays (1 vs. 0 days) were observed in the emergency patient group. Three hernia recurrences were identified in elective surgery patients and 2 cases in the emergency surgery group (p = 0.647).

### Multivariable analysis for major post-operative morbidity and 30-day mortality

The multivariable analysis revealed emergency surgery (OR 7.31; p 0.017) and CTP class C (OR 4.54; p 0.037) as unique independent risk factors for development of post-surgical morbidity (Table 2). Furthermore, it showed that emergency surgery was a unique independent risk factor for patient death (OR 10.83; p 0.028) (Table 3).

**Table 2:** Univariable and multivariable logistic regression analyses evaluating risk factors for post-operative morbidity (Clavien-Dindo Classification classes III–V)

Covariates	OR	95 % CI	p
Univariate analysis			
Urgency	9.87	2.95–37.67	0.005
CTP^b^ C	6.93	1.83–26.26	0.004
Type of hernia (umbilical vs. inguinal)	6.25	1.27-30.66	0.024
CTP^b^ A	0.09	0.01-0.74	0.025
Poorly controlled ascites	2.76	0.87-8.72	0.083
Mesh repair	0.37	0.10-1.31	0.124
Pre-operative MELD^a^ ≥ 20	3.08	0.67-14.08	0.147
Age >60 years	1.67	0.52-5.34	0.388
Combined surgery	1.82	0.28-12.00	0.533
Male gender	1.04	0.24-4.48	0.961
Multivariate analysis*			
Urgency	7.31	1.42-37.61	0.017
CTP^b^ C	4.54	1.09-18.84	0.037

Abbreviations: *a-MELD* model for end-stage liver disease, *b-CTP* Child Turcotte Pugh

*Hosmer-Lemeshow test: 0.873 (P value: 0.646)

**Table 3:** Univariable and multivariable logistic regression analyses evaluating risk factors for post-operative (within 30 days) mortality

Covariates	OR	95 % CI	p
Univariate analysis			
Urgency	10.83	1.29-91.09	0.028
Pre-operative MELD^a^ ≥ 20	4.35	1.03-18.45	0.049
Mesh repair	0.28	0.06-1.45	0.129
CTP^b^ C	2.60	0.63-10.82	0.188
Combined surgery	3.48	0.51-23.89	0.204
Poorly controlled ascites	1.96	0.52-7.31	0.317
Age > 60 years	1.94	0.51-7.37	0.328
Male gender	0.99	0.18-5.37	0.989
CTP^b^ A*	-	-	-
Type of hernia (umbilical vs. inguinal)*	-	-	-
Multivariate analysis			
Urgency	10.83	1.29-91.09	0.028

Abbreviations: a-MELD, model for end-stage liver disease; b-CTP, Child Turcotte Pugh

*phenomenon of singularity: no cases of death-

## Discussion

Cirrhotic patients have higher incidences of abdominal wall complications. Moreover, the presence of abdominal hernias has a major impact on quality of life in this patient population [9–12, 14].

The indication for surgical repair of abdominal wall hernias in cirrhotic patients remains a controversial issue. No high-quality, prospective studies have been performed to address this important question. The delay in hernia surgery is also directly related to an increased risk of life-threatening complications requiring emergency repair. Our retrospective study showed that emergently treated patients have a significantly higher morbidity and mortality. In contrast, elective surgery was not only successful in most of the patients but this strategy had almost no associated mortality.

Some reports indicate that adequate preparation of cirrhotic patients with control of ascites and improved nutritional status allows for more successful elective hernia repairs [9–12, 14, 15].

Elective hernia repair in patients with concomitant ascites may be controversial but it is not a new concept. In the early 1990s Hurst et al. [16] advocated that elective inguinal hernia repair can be performed safely in selected patients with ascites and achieving an acceptable recurrence rate is possible. In a prospective study of 30 patients with liver cirrhosis, Eker et al. [17] demonstrated successful elective repair of umbilical hernia when ascites at the time of surgery was present. After a median follow-up period of 25 months, 2 patients died (7 %), although the mortality was not related to the surgical procedure. Furthermore, Hur et al. [18] showed no operation-related mortality in 22 cirrhotic patients with ascites and inguinal hernia after undergoing elective hernia repair. McKay et al. [19] extensively reviewed medical literature regarding umbilical hernia repair in cirrhotic patients and suggested, in spite of inherent limitations in the study type, that early repair of hernias in patients with cirrhosis and ascites is a safe option for select patients and may help avoid increased morbidity and mortality associated with a possible emergency repair in the future.

The CTP classification is frequently used to identify the severity of liver disease and correlates reasonably well with post-operative morbidity and mortality in cirrhotic patients. More recently, however, the MELD score has become a more objective means to evaluate the surgical risk in cirrhotic patients. Liver disease severity in our patient cohort corresponded to a MELD score of 14. This number is of importance because it has been shown that numbers above the 8 to 14 range predicts poor surgical outcomes [20, 21]. This has been recently validated in the setting of cardiovascular, orthopaedic, and abdominal surgeries in cirrhotic patients [22, 23]. Additionally, abdominal or gastrointestinal interventions have possibly the worst outcomes when the MELD score is above 15 [24].

Concerning the timing to hernia repair in cirrhotic patients, our study is much in favour of an elective approach in selected cases. Emergency surgery was strongly associated with a higher incidence of morbidity and mortality, whereas elective surgery was successful in most patients and did not lead to serious liver-related complications. This result would be expected since there were more patients with refractory ascites and stratified as CTP B or C among emergency surgery. Interestingly, after a multivariable analysis emergency hernia surgery was the unique independent risk factor for both major morbidity and mortality and was an even stronger risk-factor than MELD scoring and CTP classification. Few data are available considering emergency repair as a major risk factor. Maniatis reported a mortality rate of 2 % after cases of elective umbilical hernia operations, which is in contrast to a 14 % mortality rate after emergency operations [25].

Poorly controlled ascites was frequently associated with emergency surgery, but it was not a significant risk factor in multivariable analysis to death or major complication (Clavien III–IV) in this study. Refractory ascites represents a direct cause of a complicated hernia (i.e. increases in abdominal pressure, skin ulceration necrosis, skin perforation, and ascites leaks) leading emergency hernia repair. Moreover, refractory ascites is not related to any allocation benefit and, in patients with low MELD scores, long transplant list waiting times are observed as well as a consequent increase in the rate of complications resulting in the need for emergency surgery.

We feel that these patients should be treated in a specialized surgical centre with extensive liver transplant experience as well as the specialized intensive and surgical care units found at such a centre. It is clear that smaller hospitals with less experience in the management of complicated liver patients presenting many times with large abdominal wall hernias do not have favourable outcomes after surgical procedures, especially emergency operations. The high morbidity and mortality of surgery in advanced cirrhotics drives many of these patients either directly or shortly after hernia repair to LT as a consequence of liver insufficiency progression. These factors make it very difficult to prospectively recruit a significant number of patients at one single centre to appropriately study outcomes of elective abdominal wall surgery in advanced cirrhotic patients.

While we believe this study has significant findings, it is not without some limitations. First, this study has a retrospective design. However, it is important to highlight that most reported studies are retrospective case series only and our study represents one of the largest experiences reported in this field. Secondly, the patient selection may be biased since it could be expected that sicker patients would not be candidates for elective hernia repair. This study aimed however, not to compare elective versus emergency hernia repairs, but to identify possible risk factors of worse outcomes in cirrhotic patients with hernia. Finally, the small patient sample reduced the ability to construct stronger statistical models using more covariates for the analysis. This limit can be easily detected when observing the large confidence intervals reported in our multivariable analyses. However, a rigorous statistical approach was adopted with the intent to select the best covariates. A preliminary univariate analysis was performed and co-linearity phenomena were avoided by testing the validity of the models. Moreover, significantly lower morbidity and mortality rates as well as shorter hospital stays were clearly in favour of the elective management of these patients.

It also became evident that the major component of success of this surgical procedure, especially when performed in an emergency situation, relates to the peri-operative management of both ascites and renal insufficiency. Advanced cirrhotic patients with major abdominal wall hernias should therefore be preferentially referred to specialised centres which also offer a liver (transplantation) unit. Given our results, we suggest lab MELD scores to be considered a useful and objective tool to further refine the therapeutic algorithm of abdominal wall hernia repair in cirrhotic patients.

We do not advocate elective surgical treatment to all cirrhotic patients, but we identified emergency surgery as a factor of higher morbidity and post-operative mortality than CTP C classification, uncontrolled ascites or high MELD score. This retrospective study was unable to determine if cirrhotic patients with uncontrolled ascites and ventral hernia are better managed with conservative or surgical hernia treatment. Therefore, we propose that patients with refractory ascites even after optimal treatment can be eligible to elective hernia repair in strict selected cases, such as patients with symptomatic hernias, thin skin, or skin ulceration in the hernia sac.

## Conclusion

The results of this retrospective study regarding the care of abdominal wall hernias in advanced cirrhotic patients indicates higher morbidity and death rates associated with emergency surgery. This finding justifies the consideration of early elective surgery. However, preoperative ascites treatments must be optimized and patients with poorly controlled ascites should be strict to patients becoming symptomatic or show signs of possible skin necrosis and rupture. Therefore, further prospective, randomized studies comparing emergency and elective abdominal wall hernia surgery in cirrhotic patients should be aimed at definitively answering the question regarding the best timing of surgery.

## Abbreviations

CI: confidence intervals
CTP: Child-Turcotte-Pugh
IQR: inter-quartile ranges
LT: liver transplantation
MELD: Model of End Stage Liver Disease
OR: odds ratio
